# Predictors and outcomes of withholding and withdrawal of life-sustaining treatments in intensive care units in Singapore: a multicentre observational study

**DOI:** 10.1186/s40560-024-00725-3

**Published:** 2024-03-26

**Authors:** Clare Fong, Wern Lunn Kueh, Sennen Jin Wen Lew, Benjamin Choon Heng Ho, Yu-Lin Wong, Yie Hui Lau, Yew Woon Chia, Hui Ling Tan, Ying Hao Christopher Seet, Wen Ting Siow, Graeme MacLaren, Rohit Agrawal, Tian Jin Lim, Shir Lynn Lim, Toon Wei Lim, Vui Kian Ho, Chai Rick Soh, Duu Wen Sewa, Chian Min Loo, Faheem Ahmed Khan, Chee Keat Tan, Roshni Sadashiv Gokhale, Chuin Siau, Noelle Louise Siew Hua Lim, Chik-Foo Yim, Jonathen Venkatachalam, Kumaresh Venkatesan, Naville Chi Hock Chia, Mei Fong Liew, Guihong Li, Li Li, Su Mon Myat, Zena Zena, Shuling Zhuo, Ling Ling Yueh, Caroline Shu Fang Tan, Jing Ma, Siew Lian Yeo, Yiong Huak Chan, Jason Phua, for SICM-NICER group

**Affiliations:** 1 FAST and Chronic Programmes, Alexandra Hospital, 378 Alexandra Road, Singapore, 159964 Singapore; 2 Division of Respiratory and Critical Care Medicine, Department of Medicine, National University Hospital, 1E Kent Ridge Road, Singapore, 119228 Singapore; 3 Department of Respiratory and Critical Care Medicine, Tan Tock Seng Hospital, 11 Jalan Tan Tock Seng, Singapore, 308433 Singapore; 4 Department of Anaesthesiology, Intensive Care and Pain Medicine, Tan Tock Seng Hospital, 11 Jalan Tan Tock Seng, Singapore, 308433 Singapore; 5 Cardiac Intensive Care Unit, Department of Cardiology, Tan Tock Seng Hospital, 11 Jalan Tan Tock Seng, Singapore, 308433 Singapore; 6 Department of Neurology, Tan Tock Seng Hospital, 11 Jalan Tan Tock Seng, Singapore, 308433 Singapore; 7 Cardiothoracic ICU, Department of Cardiac, Thoracic and Vascular Surgery, National University Heart Centre, 1E Kent Ridge Road, Singapore, 119228 Singapore; 8 Department of Anaesthesia, National University Hospital, 5 Lower Kent Ridge Road, Singapore, 119074 Singapore; 9 Department of Cardiology, National University Heart Centre, 1E Kent Ridge Road, Singapore, 119228 Singapore; 10 Department of Medicine, National University of Singapore, 1E Kent Ridge Road, Singapore, 119228 Singapore; 11 Pre-Hospital and Emergency Research Center, Duke-NUS Medical School, 8 College Rd, Singapore, 16985 Singapore; 12 Department of Intensive Care Medicine, Sengkang General Hospital, 110 Sengkang East Way, Singapore, 544886 Singapore; 13 Department of Surgical Intensive Care, Singapore General Hospital, Outram Road, Singapore, 169608 Singapore; 14 Department of Anaesthesiology, Singapore General Hospital, Outram Road, Singapore, 169608 Singapore; 15 Department of Respiratory and Critical Care Medicine, Singapore General Hospital, Outram Road, Singapore, 169608 Singapore; 16 Department of Intensive Care Medicine, Ng Teng Fong General Hospital, 1 Jurong East Street 21, Singapore, 609606 Singapore; 17 Department of Intensive Care, Changi General Hospital, 2 Simei Street 3, Singapore, 529889 Singapore; 18 Department of Respiratory and Critical Care Medicine, Changi General Hospital, 2 Simei Street 3, Singapore, 529889 Singapore; 19 Department of Anaesthesia and Surgical Intensive Care, Changi General Hospital, 2 Simei Street 3, Singapore, 529889 Singapore; 20 Department of Women’s Anaesthesia, KK Women’s and Children’s Hospital, 100 Bukit Timah Road, Singapore, 229899 Singapore; 21 Department of Respiratory and Critical Care Medicine, Khoo Teck Puat Hospital, 90 Yishun Central, Singapore, 768828 Singapore; 22 Department of Anaesthesia, Khoo Teck Puat Hospital, 90 Yishun Central, Singapore, 768828 Singapore; 23 Yong Loo Lin School of Medicine, 10 Medical Dr, Singapore, 117597 Singapore; 24 Lee Kong Chian School of Medicine, 11 Mandalay Rd, Singapore, 308232 Singapore; 25 Department of Intensive Care Unit Operations, Tan Tock Seng Hospital, 11 Jalan Tan Tock Seng, Singapore, 308433 Singapore; 26 Division of Nursing, KK Women’s and Children’s Hospital, 100 Bukit Timah Road, Singapore, 229899 Singapore; 27 Biostatistics Unit, Yong Loo Lin School of Medicine, National University of Singapore, 1E Kent Ridge Road, Singapore, 119228 Singapore

**Keywords:** Intensive care, Life-sustaining treatment, Withholding, Withdrawal

## Abstract

**Background:**

Clinical practice guidelines on limitation of life-sustaining treatments (LST) in the intensive care unit (ICU), in the form of withholding or withdrawal of LST, state that there is no ethical difference between the two. Such statements are not uniformly accepted worldwide, and there are few studies on LST limitation in Asia. This study aimed to evaluate the predictors and outcomes of withholding and withdrawal of LST in Singapore, focusing on the similarities and differences between the two approaches.

**Methods:**

This was a multicentre observational study of patients admitted to 21 adult ICUs across 9 public hospitals in Singapore over an average of three months per year from 2014 to 2019. The primary outcome measures were withholding and withdrawal of LST (cardiopulmonary resuscitation, invasive mechanical ventilation, and vasopressors/inotropes). The secondary outcome measure was hospital mortality. Multivariable generalised mixed model analysis was used to identify independent predictors for withdrawal and withholding of LST and if LST limitation predicts hospital mortality.

**Results:**

There were 8907 patients and 9723 admissions. Of the former, 80.8% had no limitation of LST, 13.0% had LST withheld, and 6.2% had LST withdrawn. Common independent predictors for withholding and withdrawal were increasing age, absence of chronic kidney dialysis, greater dependence in activities of daily living, cardiopulmonary resuscitation before ICU admission, higher Acute Physiology and Chronic Health Evaluation (APACHE) II score, and higher level of care in the first 24 h of ICU admission. Additional predictors for withholding included being of Chinese race, the religions of Hinduism and Islam, malignancy, and chronic liver failure. The additional predictor for withdrawal was lower hospital paying class (with greater government subsidy for hospital bills). Hospital mortality in patients without LST limitation, with LST withholding, and with LST withdrawal was 10.6%, 82.1%, and 91.8%, respectively (*p* < 0.001). Withholding (odds ratio 13.822, 95% confidence interval 9.987–19.132) and withdrawal (odds ratio 38.319, 95% confidence interval 24.351–60.298) were both found to be independent predictors of hospital mortality on multivariable analysis.

**Conclusions:**

Differences in the independent predictors of withholding and withdrawal of LST exist. Even after accounting for baseline characteristics, both withholding and withdrawal of LST independently predict hospital mortality. Later mortality in patients who had LST withdrawn compared to withholding suggests that the decision to withdraw may be at the point when medical futility is recognised.

**Supplementary Information:**

The online version contains supplementary material available at 10.1186/s40560-024-00725-3.

## Background

Decisions to limit life-sustaining treatments (LST), through withholding or withdrawing are commonly made in the intensive care unit (ICU) [1]. Despite recommendations by several critical care societies, there remains substantial variation in the practices surrounding LST limitations [2]. These decisions may be influenced by factors including age and pre-existing severe comorbidities, severity of the acute illness and sociocultural beliefs [3]. While clinical practice guidelines state that there is no ethical difference between withholding and withdrawal of LST [4–8], this view is not uniformly accepted across the world, especially in Asia. To some, withdrawal of therapy is seen as less acceptable [9, 10] because it is perceived as an 'action' rather than the passive 'omission' of withholding [11]. In a survey of physicians who managed patients in the ICU in 16 Asian countries and regions, 74.5% believed that withholding and withdrawal were ethically different [12].

There is a paucity of data regarding the predictors of and outcomes after limitation of LST in Asia, despite it being the world’s most populous continent [13]. Singapore, which is a microcosm of high-income Asian economies with multiple religions and cultures, offers us a unique opportunity to analyse this complex topic. Using the National Intensive Care Unit Repository, a national database, we aim to evaluate the predictors and outcomes of withholding and withdrawal of LST, with a focus on the similarities and differences between the two approaches.

## Methods

### Participants

This was a multicentre observational study of all patients admitted to adult ICUs across all public hospitals in Singapore over an average of three months per year from 2014 to 2019, using de-identified data from the National Intensive Care Unit Repository (Additional file 1: Tables S1 and S2). All patients aged 21 years or older admitted to the participating ICUs were eligible. Patients were followed until death or discharge from the hospital.

### Ethics

We obtained institutional research ethics board approval for this study (Domain Specific Review Board reference number: 2021/00887). No specific funding was received.

### Variables

Data coordinators collected data on the following baseline characteristics from both electronic and paper medical records using standardised case report forms: patient demographics including age, sex, race, religion, severe comorbidities, independence of activities of daily living, any cardiopulmonary resuscitation (CPR) within 24 h prior to ICU admission, severity of illness using the Acute Physiology and Chronic Health Evaluation (APACHE) II score, and hospital paying class (which categorises patients according to the amount of government subsidy for their hospital bills). We also recorded the overall level of care in general, and the specific types and amount of organ support provided in particular. Level 3 care meant advanced respiratory monitoring and support such as mechanical ventilation, or support for two or more organ system dysfunctions (excluding gastrointestinal support). Level 2 care meant monitoring and support for one organ system dysfunction (excluding gastrointestinal support), or basic respiratory and basic cardiovascular monitoring and support, or extended post-surgical care. Level 1 care meant no organ support but a greater degree of observation and monitoring than Level 0 (e.g. hourly or two hourly monitoring of vital signs). Level 0 care meant no organ support and normal general ward care (i.e. four hourly or less frequent vital signs monitoring) [14].

The primary outcome measures were withholding and withdrawal of LST. We defined withholding of LST as a do-not-resuscitate (DNR) order and/or an order not to start invasive mechanical ventilation and/or vasopressors and inotropes even if otherwise clinically indicated, on the grounds of lack of benefit to the patient, regardless of whether invasive mechanical ventilation and/ or vasopressors and inotropes were eventually needed [3]. We defined withdrawal of LST as the cessation of otherwise clinically indicated invasive mechanical ventilation and/or continuous infusions of vasopressors and inotropes during the ICU stay on the same grounds. We considered all patients who had both withholding and withdrawal orders as belonging to the withdrawal group. Given the aim of comparing characteristics associated with withholding and withdrawal of LST, each admission including readmissions was considered separately. Thus, the classification of withholding and withdrawal of LST applied to orders specific to each admission.

The secondary outcome measure was hospital mortality. We also recorded ICU mortality, hospital and ICU length of stay, and hospital discharge destinations.

### Statistical analysis

We compared baseline patient characteristics, treatments, and outcomes between three LST groups: (1) no limitation, (2) withholding, and (3) withdrawal. We displayed categorical variables as frequencies and percentages and made comparisons with the Chi-squared test or Fisher’s exact test, where appropriate; normally distributed continuous variables as means and standard deviations and made comparisons with the one-way analysis of variance (ANOVA) test; and non-normally distributed continuous variables as medians and interquartile ranges and made comparisons with the Kruskal–Wallis test. We used two multivariable generalised linear mixed models (GLMM) to identify among the above-stated baseline characteristics the independent predictors of LST limitation: in the first analysis, we included patients with no limitation and those with withholding of LSTs, with the former being the dependent variable; in the second analysis, we included patients with no limitation and those with withdrawal of LSTs, with the former being the dependent variable. We defined individual ICUs as random effects to account for the differences and nesting effects of ICUs. We also used GLMM to evaluate the independent association of withholding and withdrawal of LSTs with hospital mortality: variables included in this model included the withholding and withdrawal of LSTs, baseline characteristics, and level of care in the first 24 h of ICU admission. We performed Kaplan–Meier survival analysis between the three LSTs groups. We used SPSS version 20.0 (IBM Corp, Armonk, NY, USA) to analyse the data, and we considered a p value of less than 0.05 as statistically significant.

## Results

In total, data were collected from 21 ICUs across 9 hospitals and included 8907 unique patients and 9723 admissions. Of the 8907 patients, 7197 (80.8%) had no limitation of LST, 1159 (13.0%) had LST withheld, and 551 (6.2%) had LST withdrawn (Additional file 1: Fig. S1). Specifically, 1676 (18.8%) had DNR orders, 186 (2.1%) had invasive mechanical ventilation withheld, 160 (1.8%) had vasopressors/inotropes withheld, 495 (5.6%) had invasive mechanical ventilation withdrawn, and 164 (1.8%) had vasopressors/inotropes withdrawn (Additional file 1: Figs. S2, S3, and S4). Of the 9723 admissions, 7991 (82.2%) had no limitation of LST, 1181 (12.1%) had LST withheld, and 551 (5.7%) had LST withdrawn. Baseline characteristics are shown in Table 1.

**Table 1:** Baseline characteristics of each admission

Characteristics	No limitation (*n* = 7991)	Withholding (*n* = 1181)	Withdrawal (*n* = 551)	*P* value
Demographics, n (%)				
Age, mean ± SD	61.6 ± 14.9	69.1 ± 13.7	67.0 ± 14.1	< 0.001
Female sex	2881 (36.1)	446 (37.8)	203 (36.8)	0.503
Race*				0.048
Chinese	5392 (67.6)	842 (71.5)	375 (68.2)	
Malay	1206 (15.1)	172 (14.6)	79 (14.4)	
Indian	728 (9.1)	99 (8.4)	50 (9.1)	
Others	653 (8.2)	65 (5.5)	46 (8.4)	
Religion				0.069
Buddhism	2589 (32.4)	389 (32.9)	169 (30.7)	
Christianity	876 (11)	153 (13)	50 (9.1)	
Hinduism	377 (4.7)	61 (5.2)	31 (5.6)	
Islam	1338 (16.7)	195 (16.5)	80 (14.5)	
Sikhism	39 (0.5)	5 (0.4)	3 (0.5)	
Taoism	242 (3)	44 (3.7)	26 (4.7)	
Others	74 (0.9)	8 (0.7)	3 (0.5)	
None stated	2456 (30.7)	326 (27.6)	189 (34.3)	
Severe comorbidities, *n* (%)				
Chronic kidney dialysis	638 (8)	127 (10.8)	49 (8.9)	0.005
Malignancy^a^	419 (5.2)	123 (10.4)	41 (7.4)	< 0.001
Immunocompromised^b^	417 (5.2)	105 (8.9)	40 (7.3)	< 0.001
Chronic liver failure^c^	215 (2.7)	68 (5.8)	17 (3.1)	< 0.001
Severe cardiovascular disease^d^	113 (1.4)	39 (3.3)	12 (2.2)	< 0.001
Severe respiratory disease^e^	80 (1)	25 (2.1)	10 (1.8)	0.002
Activities of daily living^f^, *n* (%)				< 0.001
Independent	7021 (87.9)	907 (76.8)	433 (78.6)	
Partially dependent	724 (9.1)	190 (16.1)	87 (15.8)	
Totally dependent	246 (3.1)	84 (7.1)	31 (5.6)	
CPR 24 h before ICU admission, *n* (%)	374 (4.7)	270 (22.9)	170 (30.9)	< 0.001
APACHE II score, mean ± SD	17.5 ± 7.4	27.2 ± 9	25.6 ± 8.1	< 0.001
Hospital paying class, *n* (%)				0.001
A and B1^g^	855 (10.7)	103 (8.7)	34 (6.2)	
B2 and C^h^	7136 (89.3)	1078 (91.3)	517 (93.8)	

*SD* standard deviation, *CPR* cardiopulmonary resuscitation, *ICU* intensive care unit, *APACHE* Acute Physiology and Chronic Health Evaluation

Given the aim of comparing characteristics associated with withholding and withdrawal of life-sustaining treatments, each admission including readmissions is considered separately; thus, classification of withholding and withdrawal applies to orders specific to each admission

^a^^−^^h^Please see Additional file 1: Table S5 for the definition of these baseline characteristics

*Data missing for 16 patients for race

Patients with LST limitation (withholding or withdrawal) had more days on invasive mechanical ventilation, more number of specific organs supported including respiratory, cardiovascular, gastrointestinal, neurological, renal, and liver support, and a higher overall level of care (Table 2).

**Table 2:** Organ support and level of care of each admission

Treatment	No limitation (*n* = 7991)	Withholding (*n* = 1181)	Withdrawal (*n* = 551)	*P* value
Organ support modalities, *n* (%)				
IMV days, median (IQR)	2 (0–3)	3 (1–7)	5 (3–9)	< 0.001
Respiratory				< 0.001
Advanced support^a^	5782 (72.4)	1033 (87.5)	545 (98.9)	
Basic support^b^	655 (8.2)	78 (6.6)	4 (0.7)	
No support	1554 (19.5)	70 (5.9)	2 (0.4)	
Cardiovascular				< 0.001
Advanced support^c^	1912 (23.9)	512 (43.4)	168 (30.5)	
Basic support^d^	5158 (64.6)	575 (48.7)	353 (64.1)	
No support	921 (11.5)	94 (8)	30 (5.4)	
Gastrointestinal^e^	3929 (49.2)	724 (61.3)	423 (76.8)	< 0.001
Neurological^f^	836 (10.5)	126 (10.7)	131 (23.8)	< 0.001
Renal^g^	1268 (15.9)	329 (27.9)	106 (19.2)	< 0.001
Liver^h^	104 (1.3)	31 (2.6)	10 (1.8)	0.002
Dermatological^i^	11 (0.1)	4 (0.3)	1 (0.2)	0.190
Number of organs supported, median (IQR)	2 (2–2)	2 (2–3)	2 (2–3)	< 0.001
Percentage of days spent at level of care, median (IQR)				
Level 3^j^	75 (40–100)	100 (88–100)	100 (95–100)	< 0.001
Level 2^ k^	17 (0–50)	0 (0–10)	0 (0–3)	< 0.001
Level 1^ l^	0 (0–0)	0 (0–0)	0 (0–0)	< 0.001
Level 0^ m^	0 (0–0)	0 (0–0)	0 (0–0)	0.028
Care in first 24 h of ICU admission, n (%)				< 0.001
Level 3	5437 (68)	963 (81.5)	487 (88.4)	
Level 2	1500 (18.8)	101 (8.6)	7 (1.3)	
Level 1 or Level 0	1054 (13.2)	117 (9.9)	57 (10.3)	
Care at ICU discharge, *n* (%)*	(*n* = 7591)	(*n* = 394)	(*n* = 222)	< 0.001
Level 3	574 (7.6)	36 (9.1)	3 (1.4)	
Level 2	3967 (52.3)	177 (44.9)	85 (38.3)	
Level 1	2771 (36.5)	169 (42.9)	122 (55)	
Level 0	279 (3.7)	12 (3.1)	12 (5.4)	

*IMV* invasive mechanical ventilation, *IQR* interquartile range, *ICU* intensive care unit

Given the aim of comparing characteristics associated with withholding and withdrawal of life-sustaining treatments, each admission including readmissions is considered separately; thus, classification of withholding and withdrawal applies to orders specific to each admission

^a^^−^^m^Please see Additional file 1: Table S6 for the definition of organ support and level of care

*400, 787, 329 patients in the no limitation group, withholding and withdrawal group, respectively, were deceased

Common independent predictors for both withholding and withdrawal of LST using GLMM were greater age, no chronic dialysis, greater dependence in activities of daily living, cardiopulmonary resuscitation 24 h before ICU admission, higher APACHE II score, and higher level of care in the first 24 h of ICU admission. Additional predictors for withholding of LST included being of a Chinese race, the religions of Hinduism and Islam, malignancy, and chronic liver failure. As for withdrawal of LST, the unique predictor identified was lower hospital paying class (with greater government subsidy for hospital bills) (Table 3 and Additional file 1: Table S3).

**Table 3:** Independent predictors of withholding and withdrawal of life-sustaining treatments

Withholding of life-sustaining treatments^	OR (95% CI)	*P* value
Age	1.020 (1.014–1.026)	< 0.001
Race		0.031
Chinese	Reference	
Malay	0.652 (0.460–0.924)	0.016
Indian	0.663 (0.450–0.976)	0.037
Others	0.674 (0.475–0.957)	0.027
Religion		0.047
No religion	Reference	
Buddhism	0.901 (0.739–1.099)	0.305
Christianity	1.156 (0.897–1.490)	0.262
Hinduism	1.628 (1.003–2.6430	0.049
Islam	1.588 (1.123–2.246)	0.009
Sikhism	1.349 (0.471–3.865)	0.578
Taoism	0.803 (0.538–1.198)	0.283
Others	0.762 (0.338–1.716)	0.511
Chronic kidney dialysis	0.728 (0.577–0.919)	0.008
Malignancy	1.435 (1.095–1.879)	0.009
Chronic liver failure	1.437 (1.044–1.979)	0.026
Activities of daily living		< 0.001
Independent	Reference	
Partially dependent	1.300 (1.058–1.597)	0.012
Totally dependent	1.899 (1.408–2.560)	< 0.001
CPR 24 h before ICU admission	2.360 (1.916–2.907)	< 0.001
APACHE II score	1.114 (1.103–1.124)	< 0.001
Care in first 24 h of ICU admission		0.018
Level 3	Reference	
Level 2	0.746 (0.588–0.945)	0.015
Level 1 and Level 0	0.803 (0.633–1.020)	0.072
Withdrawal of life-sustaining treatments^	OR (95% CI)	*P* value
Age	1.015 (1.008–1.023)	< 0.001
Chronic kidney dialysis	0.607 (0.427–0.865)	0.006
Activities of daily living		0.044
Independent	Reference	
Partially dependent	1.366 (1.027–1.816)	0.032
Totally dependent	1.410 (0.908–2.190)	0.126
CPR 24 h before ICU admission	5.409 (4.216–6.940)	< 0.001
APACHE II score	1.088 (1.074–1.102)	< 0.001
Hospital paying class		0.045
B2 and C	Reference	
A and B1	0.665 (0.447–0.990)	0.045
Care in first 24 h of ICU admission		< 0.001
Level 3	Reference	
Level 2	0.087 (0.040–0.185)	< 0.001
Level 1 and Level 0	0.673 (0.488–0.926)	0.015

*OR* odds ratio, *CI* confidence intervals, *CPR* cardiopulmonary resuscitation, *ICU* intensive care unit, *APACHE* Acute Physiology and Chronic Health Evaluation

^Only variables found to be independently associated with withholding and withdrawal of life-sustaining treatments, when compared to no limitation, on generalised mixed model analyses are shown. The full table can be found in Additional File 1: Table S3

**Fig. 1:**
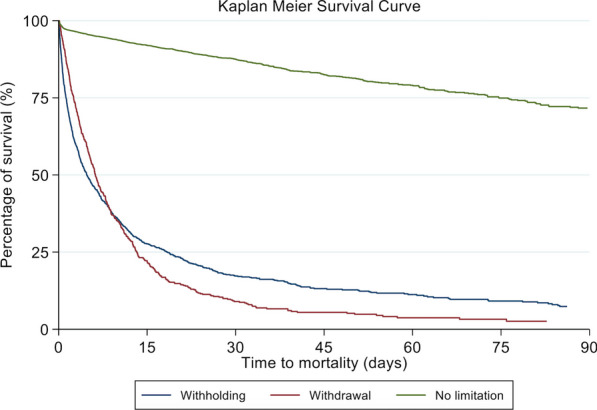
Kaplan–Meier survival curve for three groups: no limitation, withholding, withdrawal. Patients discharged alive were excluded from the Kaplan Meier survival analysis and a cut-off time of 90 days was used

Among 2217 hospital deaths, 34.2% occurred without LST limitation, 42.9% with LST withholding, and 22.8% with LST withdrawal. Hospital mortality was higher among patients with LST withholding (82.1%) and LST withdrawal (91.8%) than those without LST limitation (10.6%) (p < 0.001) (Table 4). The no limitation group had the longest median hospital length of stay whilst the withdrawal group had the longest median ICU length of stay. A higher percentage of patients in the no limitation group were discharged home. Using GLMM, withholding (odds ratio 13.822, 95% confidence interval 9.987–19.132, p < 0.001) and withdrawal (odds ratio 38.319, 95% confidence interval 24.351–60.298, p < 0.001) of LST were both found to be independent predictors of hospital mortality (Additional file 1: Table S4). Kaplan–Meier survival analysis at 90 days showed a lower percentage survival for those with either withholding or withdrawal of LST (Fig. 1).

**Table 4:** Outcomes of patients

Outcomes	No limitation (*n* = 7197)	Withholding (*n* = 1159)	Withdrawal (*n* = 551)	*P* value
Hospital mortality, *n* (%)	759 (10.6)	952 (82.1)	506 (91.8)	< 0.001
ICU mortality, *n* (%)	358 (5)	782 (67.5)	329 (59.7)	< 0.001
Hospital days, median (IQR)	14 (7.1–29.9)	10.1 (2.7–24.1)	8.6 (3.4–18.2)	< 0.001
ICU days, median (IQR)	1.9 (0.9–4)	2.7 (1–6.9)	4.7 (2.1–8.9)	< 0.001
Hospital discharge destination for survivors, *n* (%)	(*n* = 6438)	(*n* = 207)	(*n* = 45)	< 0.001
Home	5038 (78.3)	134 (64.7)	25 (55.6)	
Community rehabilitation hospital	780 (12.1)	32 (15.5)	7 (15.6)	
Long-term nursing home	251 (3.9)	27 (13)	10 (22.2)	
Another acute hospital’s ICU/HDU	180 (2.8)	3 (1.5)	1 (2.2)	
Another acute hospital’s general ward	152 (2.4)	5 (2.4)	2 (4.4)	
Hospice	21 (0.3)	5 (2.4)	0 (0)	
Others	16 (0.3)	1 (0.5)	0 (0)	

*ICU* intensive care unit*, IQR* interquartile range*, HDU* high-dependency unit

Given the aim of comparing the secondary outcome of hospital mortality with the primary outcomes of withholding and withdrawal of life-sustaining treatments, numbers refer to patients rather than ICU admissions and readmissions. Patients are categorised as receiving withholding or withdrawal orders as long as the orders were made in at least one admission during the hospital stay. Hospital outcomes refer to those of the entire hospital stay. ICU outcomes refer to the ICU admission where withholding or withdrawal orders were made for patients with multiple ICU admissions within the hospital stay

## Discussion

In total, 19.2% of patients in our study had LST limitation (13.0% withheld and 6.2% withdrawn, respectively). While a number of factors were found to be common independent predictors of both withholding and withdrawal of LST, there were additional independent predictors specifically for withholding and for withdrawal of LST. Both withholding and withdrawal of LST were independent predictors of hospital mortality.

International differences in practices of LST limitation are striking [2, 3]. Considering all ICU patients, the proportion with LST limitation in our study (19.2%) was higher than the international average of 11.8% seen in the ETHICUS-2 study which surveyed 199 ICUs in 36 countries in 2015 and 2016 and the international average of 13.2% seen in the Intensive Care Over Nations (ICON) study which surveyed 730 ICUs in 84 countries in 2012 [15, 16]. On the other hand, considering only patients who ultimately died in hospital, the proportion with LST limitation in our study (65.8%) was lower than the international average of 76.6% in the ETHICUS-2 study but higher than the international average of 39.5% in the ICON study. Notably, our study included DNR orders as withholding of LST, just as the ETHICUS-2 study did, while the ICON study did not specify details. Focusing on Asia, the 19.2% with LST limitation in our study was between the average of 10.9% across China, Hong Kong, India, Japan, South Korea, and Thailand in the ETHICUS-2 study and the average of 30% across China, Indonesia, Japan, Malaysia, the Philippines, Singapore, Taiwan, and Thailand in the ICON study. In the Asian Collaboration for Medical Ethics (ACME) study of attitudes towards end-of-life care in the ICU in Asia, ICU physicians in Singapore tended to withhold and withdraw LST more than many other countries, possibly due to a combination of factors including a Western influence on education in a high-income economy [3, 12]. Indeed, decisions to limit LST are less frequently reported in countries with low or lower-to-middle gross national income [16].

Most studies do not distinguish between predictors of withholding and withdrawal of LST. Common independent predictors of both withholding and withdrawal of LST in our study were greater age, no chronic kidney dialysis, greater dependence in activities of daily living, cardiopulmonary resuscitation 24 h before ICU admission, higher APACHE II score, and higher level of care in the first 24 h of ICU admission. Other than absence of chronic kidney dialysis, this is similar to other factors identified in Europe, the United States, and Australia, where patients with LST limitation were older [17–21], had severe comorbidities [8, 22], poorer baseline functional status [16], and were more severely ill [19, 20, 23]. In an international ethics round table consensus statement, factors considered important in deciding to limit LST were an expected survival of less than 1–3 months, multiple organ failure, very severe brain injury, and the health care team's opinion that the patient is receiving non-beneficial therapy or has a nonsurvivable injury [24]. What is surprising in our study is that chronic kidney dialysis was not a predictor for limitation of LST. This may be because chronic kidney dialysis patients are at higher risk of complications and would thus more likely be admitted to a higher acuity setting for post-procedure monitoring. In our local context, LST is typically not limited when patients undergo invasive procedures, so that adequate support can be provided in the event of any post-procedural complications. In addition, the prognosis of chronic kidney disease is uncertain and involves complex decision-making. Prior studies have shown that palliative care is often suboptimal for patients with end stage kidney disease receiving dialysis treatment, with a high rate of intensive care needs towards the end of life [25], and that most nephrologists feel unprepared to lead end-of-life care preferences discussions [26].

In our study, a higher proportion of patients had LST withheld rather than withdrawn. This is consistent with a survey performed in Asian ICUs where physicians reported that they often withheld but seldom withdrew LST at the end of life [12], and may reflect physicians’ views that the two are not the same. Importantly, our findings highlight three differences in additional predictors of withholding versus withdrawal of LST, despite experts’ assertions that the two are ethically similar [6]. First, although it is known that practices of LST limitation are driven by patient demographics and cultural differences, previous studies—which were mostly from the United States and Europe—had generally not distinguished between withholding and withdrawal [18, 24, 27, 28]. In our study, being of a Chinese race and having the religions of Hinduism or Islam independently predicted the withholding but not the withdrawal of LST. While pre-existing data in the literature suggest that the people of China were generally averse to DNR orders [29], and that Hindu and Islamic physicians in Asian ICUs were less likely to withdraw LST [30], such findings are not directly applicable to Singapore’s multicultural society. Second, our findings that malignancy and chronic liver failure independently predicted withholding but not withdrawal of LST are consistent with those of the ICON study and the international SAPS 3 database, although these studies did not differentiate the two forms of LST limitation [16, 31]. Third, lower hospital paying class (meaning more government subsidy) was independently associated with LST withdrawal but not withholding. In Singapore, while greater subsidies for hospital bills are provided for patients’ with lower monthly incomes, out-of-pocket payments are still required [32]. While previous data show that financial considerations play a significant part in decisions to withdraw LST in Asian low-middle income countries [30], our results imply that this may also apply to a high-income nation like Singapore. Taken together, the three afore-mentioned points suggest that withholding of LST is more often affected by baseline characteristics such as race, religion, and comorbidities, while withdrawal of LST is more often considered when costs accumulate as the provision of intensive care continues for longer than anticipated and when the point of medical futility is reached. As depicted in the Kaplan–Meier survival curve (Fig. 1), withholding was more strongly associated with earlier mortality, but withdrawal was more associated with later mortality.

Hospital mortality was 82.1% in our withholding group and 91.8% in our withdrawal group, compared to 71.9% and 88.5%, respectively, in the international ETHICUS-2 study and 86.4% and 92.5%, respectively, in the international SAPS 3 database [15, 31]. The mortality of patients with LST withdrawal was higher than that of patients with LST withholding on univariable analysis. Both were higher than that of patients with no LST limitation. This is unsurprising, since physicians often reserve LST limitation for patients with the gravest prognosis. What is more instructive, however, is that even after accounting for baseline characteristics including severity of illness and level of care in the first 24 h of ICU admission through multivariable GLMM analysis, withholding (odds ratio 13.822) and withdrawal (odds ratio 38.319) were still significantly associated with mortality. While the presence of hidden confounders such as illness severity must be considered, the high odds ratios suggest that hospital survival may have been possible had LST not been limited.

Another interesting finding in our study is that although a minority of patients with LST limitation survived till hospital discharge, a substantial proportion of this minority were discharged home (as opposed to another healthcare facility). 64.7% of hospital survivors who had LST withheld, and 55.6% of hospital survivors who had LST withdrawn, were discharged home. As patients were followed up only until hospital discharge, it is unknown whether these patients passed away soon after, or if a terminal discharge was arranged. In Singapore, terminal discharge is not an uncommon practice [33]. To the best of our knowledge, prior studies on limitation of LST practices report hospital survival but do not have data regarding hospital discharge destination.

Our study has several strengths. First, it was conducted in 21 ICUs across all Singaporean public hospitals over a substantial period of six years, with a large sample size. Second, there were hardly any missing data. Third, it contributes knowledge to a scientific field which is still overwhelmingly dominated by a Western perspective and under-represented by Asian countries. Fourth, it explored an important topic through a unique lens, focusing on the similarities and differences of withholding and withdrawal of LST. Our study also has several limitations. First, it was confined to a single country. Nonetheless, given Singapore’s multicultural nature, it is to our knowledge the first study to compare LST limitation practices in the ICU for several of the world’s major races and religions in one country. Second, it recruited patients over an average of three months annually, rather than across the year. This was done so as to be able to include as many ICUs as possible with the number of data coordinators available. Regardless, the findings remain representative as practice patterns were unlikely to have varied depending on the time of the year. Finally, specific information on when exactly the decisions were made to limit LST was not available, thus preventing a detailed analysis of circumstances around such decisions.

Our study has implications for future research in end-of-life care in the ICU. Investigators aiming to elucidate the predictors of LST limitation in local, regional, or international settings should clearly differentiate withholding and withdrawal, because the two are not the same. Future work should also go beyond exploring the univariable association of LST limitation with mortality to evaluating the true cause-and-effect impact of withholding as well as withdrawal of LST on short and long-term patient-centric outcomes.

## Conclusion

Differences in the independent predictors of withholding and withdrawal of LST exist. In addition, even after accounting for baseline characteristics, both withholding and withdrawal of LST independently predict hospital mortality. Later mortality in patients who had LST withdrawn compared to withholding may suggest that the decision to withdraw may be when the point of medical futility is recognised.

### Supplementary Information

**Additional file 1: Table S1.** Dates of data collection. **Table S2.** Participating intensive care units. **Table S3.** Independent predictors of withholding and withdrawal of life-sustaining treatments. **Table S4.** Independent predictors of hospital mortality. **Table S5.** Definition of baseline characteristics. **Table S6.** Definition of organ support and level of care. **Table S7.** Annual LST limitation and hospital mortality rate in Singapore. **Figure S1.** Flow diagram of patients for both invasive mechanical ventilation and vasopressors/inotropes. **Figure S2.** Flow diagram of patients for DNR order. **Figure S3.** Flow diagram of patients for invasive mechanical ventilation. **Figure S4**. Flow diagram of patients for vasopressors/inotropes.

## Data Availability

The datasets used and/or analysed during the current study are available from the corresponding author on reasonable request.

## Abbreviations

ACME: Asian Collaboration for Medical Ethics
ANOVA: Analysis of variance
APACHE: Acute Physiology and Chronic Health Evaluation
CPR: Cardiopulmonary resuscitation
DNR: Do-not-resuscitate ICU
GLMM: Generalised linear mixed models
ICON: Intensive Care Over Nations
ICU: Intensive care unit
LST: Life-sustaining treatments
