# Supplementary material for: Predictors and outcomes of withholding and withdrawal of life-sustaining treatments in intensive care units in Singapore: a multicentre observational study
Source: J Intensive Care. 2024 Mar 26;12:13. doi: 10.1186/s40560-024-00725-3 (PMC10964634; doi:10.1186/s40560-024-00725-3)
Supplement: Supplementary file 1 — Additional file 1: Table S1. Dates of data collection. Table S2. Participating intensive care units. Table S3. Independent predictors of withholding and withdrawal of life-sustaining treatments. Table S4. Independent predictors of hospital mortality. Table S5. Definition of baseline characteristics. Table S6. Definition of organ support and level of care. Table S7. Annual LST limitation and hospital mortality rate in Singapore. Figure S1. Flow diagram of patients for both invasive mechanical ventilation and vasopressors/inotropes. Figure S2. Flow diagram of patients for DNR order. Figure S3. Flow diagram of patients for invasive mechanical ventilation. Figure S4. Flow diagram of patients for vasopressors/inotropes. [file 40560_2024_725_MOESM1_ESM.docx]

**Table S1**. Dates of data collection

| **Year** | **Dates** |
| --- | --- |
| 2014 | 1 October 2014 to 31 December 2014 |
| 2015 | 1 October 2015 to 31 December 2015 |
| 2016 | 1 April 2016 to 30 June 2016 |
| 2017 | 1 October 2017 to 31 December 2017 |
| 2018 | 1 October 2018 to 31 December 2018 |
| 2019 | 1 October 2019 to 31 December 2019 |

**Table S2**. Participating intensive care units

| **Hospital** | **ICU** |
| --- | --- |
| Alexandra Hospital | Multidisciplinary ICU |
| Changi General Hospital | Medical ICU |
|  | Surgical ICU |
| Khoo Teck Puat Hospital | Medical ICU |
|  | Surgical ICU |
| KK Women’s and Children’s Hospital | Women’s ICU |
| Ng Teng Fong General Hospital | Multidisciplinary ICU |
| National University Hospital | Cardiology ICU |
|  | Cardiothoracic ICU |
|  | Medical ICU |
|  | Surgical ICU |
| Sengkang General Hospital | Multidisciplinary ICU |
| Singapore General Hospital | Burns ICU |
|  | Medical ICU |
|  | Neurosurgical ICU |
|  | Surgical ICU |
| Tan Tock Seng Hospital | Cardiology ICU |
|  | Medical ICU |
|  | Neurosurgical ICU |
|  | Neurology ICU |
|  | Surgical ICU |

*ICU* Intensive Care Unit

**Table S3**. Independent predictors of withholding and withdrawal of life-sustaining treatments

| **Withholding of life-sustaining treatments** | **OR (95% CI)** | **P value** |
| --- | --- | --- |
| Age | 1.020 (1.014-1.026) | <0.001 |
| Female sex | 0.966 (0.835-1.117) | 0.638 |
| Race |  | 0.031 |
| Chinese | Reference |  |
| Malay | 0.652 (0.460-0.924) | 0.016 |
| Indian | 0.663 (0.450-0.976) | 0.037 |
| Others | 0.674 (0.475-0.957) | 0.027 |
| Religion |  | 0.047 |
| No religion | Reference |  |
| Buddhism | 0.901 (0.739-1.099) | 0.305 |
| Christianity | 1.156 (0.897-1.490) | 0.262 |
| Hinduism | 1.628 (1.003-2.6430 | 0.049 |
| Islam | 1.588 (1.123-2.246) | 0.009 |
| Sikhism | 1.349 (0.471-3.865) | 0.578 |
| Taoism | 0.803 (0.538-1.198) | 0.283 |
| Others | 0.762 (0.338-1.716) | 0.511 |
| Chronic kidney dialysis | 0.728 (0.577-0.919) | 0.008 |
| Malignancy | 1.435 (1.095-1.879) | 0.009 |
| Immunocompromised | 1.053 (0.789-1.404) | 0.726 |
| Chronic liver failure | 1.437 (1.044-1.979) | 0.026 |
| Severe cardiovascular disease | 1.058 (0.677-1.653) | 0.804 |
| Severe respiratory disease | 1.409 (0.850-2.338) | 0.184 |
| Activities of daily living |  | <0.001 |
| Independent | Reference |  |
| Partially dependent | 1.300 (1.058-1.597) | 0.012 |
| Totally dependent | 1.899 (1.408-2.560) | <0.001 |
| CPR 24h before ICU admission | 2.360 (1.916-2.907) | <0.001 |
| APACHE II score | 1.114 (1.103-1.124) | <0.001 |
| Hospital paying class |  | 0.574 |
| B2 and C | Reference |  |
| A and B1 | 1.074 (0.836-1.380) | 0.574 |
| Care in first 24 hours of ICU admission |  | 0.018 |
| Level 3 | Reference |  |
| Level 2 | 0.746 (0.588-0.945) | 0.015 |
| Level 1 and Level 0 | 0.803 (0.633-1.020) | 0.072 |
| **Withdrawal of life-sustaining treatments** | **OR (95% CI)** | **P value** |
| Age | 1.015 (1.008-1.023) | <0.001 |
| Female sex | 0.951 (0.779-1.162) | 0.624 |
| Race |  | 0.122 |
| Chinese | Reference |  |
| Malay | 1.006 (0.656-1.543) | 0.978 |
| Indian | 0.940 (0.570-1.550) | 0.808 |
| Others | 1.555 (1.038-2.331) | 0.032 |
| Religion |  | 0.276 |
| No religion | Reference |  |
| Buddhism | 0.767 (0.583-1.010) | 0.059 |
| Christianity | 0.694 (0.477-1.009) | 0.056 |
| Hinduism | 1.199 (0.638-2.250) | 0.573 |
| Islam | 0.889 (0.575-1.375) | 0.598 |
| Sikhism | 1.165 (0.317-4.281) | 0.818 |
| Taoism | 1.145 (0.699-1.874) | 0.591 |
| Others | 0.616 (0.176-2.148) | 0.447 |
| Chronic kidney dialysis | 0.607 0.427-0.865) | 0.006 |
| Malignancy | 1.191 (0.792-1.789) | 0.401 |
| Immunocompromised | 1.149 (0.756-1.746) | 0.515 |
| Chronic liver failure | 0.904 (0.530-1.541) | 0.711 |
| Severe cardiovascular disease | 0.703 (0.338-1.459) | 0.344 |
| Severe respiratory disease | 1.215 (0.584-2.528) | 0.602 |
| Activities of daily living |  | 0.044 |
| Independent | Reference |  |
| Partially dependent | 1.366 (1.027-1.816) | 0.032 |
| Totally dependent | 1.410 (0.908-2.190) | 0.126 |
| CPR 24h before ICU admission | 5.409 (4.216-6.940) | <0.001 |
| APACHE II score | 1.088 (1.074-1.102) | <0.001 |
| Hospital paying class |  | 0.045 |
| B2 and C | Reference |  |
| A and B1 | 0.665 (0.447-0.990) | 0.045 |
| Care in first 24 hours of ICU admission |  | <0.001 |
| Level 3 | Reference |  |
| Level 2 | 0.087 (0.040-0.185) | <0.001 |
| Level 1 and Level 0 | 0.673 (0.488-0.926) | 0.015 |

*OR* odds ratio, *CI* confidence intervals, *CPR* cardiopulmonary resuscitation, *ICU* intensive care unit, *APACHE* Acute Physiology and Chronic Health Evaluation

**Table S4**. Independent predictors of hospital mortality

| **Predictors^a^** | **OR (95% CI)** | **P value** |
| --- | --- | --- |
| Limitation on LSTs |  | <0.001 |
| No limitation | Reference |  |
| Withholding | 13.822 (9.987-19.132) | <0.001 |
| Withdrawal | 38.319 (24.351-60.298) | <0.001 |
| Age | 1.010 (1.002-1.019) | 0.022 |
| Female sex | 0.775 (0.663-0.905) | 0.001 |
| Race |  | 0.533 |
| Chinese | Reference |  |
| Malay | 0.971 (0.730-1.292) | 0.842 |
| Indian | 0.961 (0.777-1.1870 | 0.711 |
| Others | 1.069 (0.803-1.425) | 0.646 |
| Religion |  | 0.002 |
| No religion | Reference |  |
| Buddhism | 0.919 (0.730-1.156) | 0.470 |
| Christianity | 0.891 (0.689-1.151) | 0.377 |
| Hinduism | 0.545 (0.370-0.782) | 0.001 |
| Islam | 0.787 (0.535-1.157) | 0.223 |
| Sikhism | 1.595 (0.901-2.822) | 0.109 |
| Taoism | 0.746 (0.452-1.231) | 0.252 |
| Others | 0.952 (0.477-1.902) | 0.890 |
| Chronic kidney dialysis | 0.552 (0.387-0.787) | 0.001 |
| Malignancy | 1.479 (1.017-2.152) | 0.041 |
| Immunocompromised | 1.071 (0.739-1.553) | 0.717 |
| Chronic liver failure | 1.066 (0.654-1.735) | 0.799 |
| Severe cardiovascular disease | 1.129 (0.736-1.731) | 0.578 |
| Severe respiratory disease | 0.726 (0.382-1.381) | 0.329 |
| Activities of daily living |  | 0.004 |
| Independent | Reference |  |
| Partially dependent | 1.644 (1.224-2.208) | 0.001 |
| Totally dependent | 0.963 (0.617-1.505) | 0.870 |
| CPR 24h before ICU admission | 2.294 (1.855-2.836) | <0.001 |
| APACHE II score | 1.100 (1.079-1.123) | <0.001 |
| Hospital paying class |  | 0.028 |
| B2 and C | Reference |  |
| A and B1 | 0.734 (0.557-09.67) | 0.028 |
| IMV days | 1.019 (1.005-1.0330 | 0.008 |
| Respiratory |  | 0.101 |
| No support | Reference |  |
| Basic support | 0.616 (0.138-2.754) | 0.526 |
| Advanced support | 0.357 (0.113-1.131) | 0.08 |
| Cardiovascular |  | <0.001 |
| No support | Reference |  |
| Basic support | 0.492 (0.105-2.300) | 0.367 |
| Advanced support | 1.116 (0.179-6.946) | 0.907 |
| Gastrointestinal | 1.022 (0.870-1.202) | 0.788 |
| Neurological | 0.532 (0.124-2.273) | 0.394 |
| Renal | 0.848 (0.225-3.191) | 0.807 |
| Liver | 0.936 (0.256-3.425) | 0.920 |
| Number of organs supported | 1.609 (0.383-6.757) | 0.516 |
| Days spent at level of care at Level 1 | 17.261 (2.379-125.215) | 0.005 |
| Days spent at level of care at Level 2 | 15.497 (2.249-106.768) | 0.005 |
| Days spent at level of care at Level 3 | 123.396 (14.516-1048.926) | <0.001 |
| Care in first 24 hours of ICU admission |  | <0.001 |
| Level 3 | Reference |  |
| Level 2 | 2.295 (1.488-3.541) | <0.001 |
| Level 1 and Level 0 | 1.507 (1.218-1.864) | <0.001 |

*LST* life-sustaining treatments*, OR* odds ratio, *CI* confidence intervals, *CPR* cardiopulmonary resuscitation, *ICU* intensive care unit, *APACHE* Acute Physiology and Chronic Health Evaluation, *IMV* invasive mechanical ventilation

Given the aim of finding the association between the secondary outcome of hospital mortality and the primary outcomes of withholding and withdrawal of life-sustaining treatments, baseline characteristics incorporated into the generalised linear mixed model refer to patients rather than ICU admissions and readmissions. Patients are categorised as receiving withholding or withdrawal orders as long as the orders were made in at least one admission during the hospital stay.

^a^ Only variables found to be independently associated with hospital mortality on generalised linear mixed model analysis are shown

**Table S5**. Definition of baseline characteristics

| **Characteristics** | **Definition** |
| --- | --- |
| Malignancy^a^ | Metastatic disease, leukaemia, lymphoma, or multiple myeloma |
| Immunocompromised^b^ | Immunosuppressive therapy, human immunodeficiency virus infection, congenital immunohumoral or cellular immune deficiency state |
| Chronic liver failure^c^ | Biopsy proven cirrhosis, portal hypertension, or episodes of hepatic encephalopathy |
| Severe cardiovascular disease^d^ | New York Heart Association functional class IV |
| Severe respiratory disease^e^ | Unable to work, has shortness of breath performing most normal activities of daily living, or home ventilation |
| Activities of daily living^f^ | Bathing, dressing, going to the toilet, moving in/out of bed/chair, continence, and eating |
| A and B1^g^ | 0-20% of hospitalisation bill subsidised by government |
| B2 and C^h^ | 50-80% of hospitalisation bill subsidised by government |

**Table S6**. Definition of organ support and level of care

| **Characteristics** | **Definition** |
| --- | --- |
| Respiratory | |
| Advanced support^a^ | Mechanical ventilation or extracorporeal respiratory support |
| Basic support^b^ | More than 50% oxygen via face mask, non-invasive ventilation, high flow nasal cannula, close observation for acute respiratory deterioration, or at least two hourly physiotherapy or suctioning to clear secretions |
| Cardiovascular | |
| Advanced support^c^ | Multiple intravenous vasoactive or rhythm controlling drugs, continuous cardiac output monitoring, intra-aortic balloon pump, or temporary cardiac pacemaker |
| Basic support^d^ | Central venous pressure or arterial pressure monitoring, or a single vasoactive or rhythm-controlling drug |
| Gastrointestinal^e^ | Enteral or parenteral nutrition |
| Neurological^f^ | Central nervous depression sufficient to compromise airway, invasive neurological monitoring, extra-ventricular drainage, continuous intravenous medication to control seizures and/or for cerebral monitoring, or therapeutic hypothermia |
| Renal^g^ | Kidney replacement therapy |
| Liver^h^ | Liver dialysis or receipt of agents for management of coagulopathy as a result of liver conditions |
| Dermatological^i^ | Major skin rashes, exfoliation, or burns (more than 30% body surface area) |
| Level of care | |
| Level 3^j^ | Advanced respiratory monitoring and support such as mechanical ventilation or monitoring; and support for two or more organ system dysfunctions (excluding gastrointestinal support) |
| Level 2^k^ | Monitoring and support for one organ system dysfunction (excluding gastrointestinal support), or basic respiratory and basic cardiovascular monitoring and support, or extended post-surgical care |
| Level 1^l^ | No organ support but greater degree of observation and monitoring than Level 0 (e.g. hourly or two hourly monitoring of vital signs) |
| Level 0^m^ | No organ support and normal general ward care (i.e. four hourly or less frequent vital signs monitoring) |

**Table S7**. Annual LST limitation and hospital mortality rate

| **Year** | **LST limitation rate** | **Hospital mortality rate** |
| --- | --- | --- |
| 2014 | 17.0% | 25.4% |
| 2015 | 20.0% | 26.3% |
| 2016 | 18.4% | 23.5% |
| 2017 | 20.2% | 24.0% |
| 2018 | 18.4% | 24.8% |
| 2019 | 20.9% | 25.9% |
| Study | 19.2% | 24.8% |

# Figure S1

**Figure S1**. Flow diagram of patients for both invasive mechanical ventilation and vasopressors/inotropes

*ICU* intensive care unit

Given the aim of comparing the secondary outcome of hospital mortality with the primary outcomes of withholding and withdrawal of life-sustaining treatments, numbers refer to patients rather than ICU admissions and readmissions. Patients are categorised as receiving withholding or withdrawal orders as long as the orders were made in at least one ICU admission during the hospital stay. ICU deaths refer to the ICU admission where withholding or withdrawal orders were made for patients with multiple ICU admissions within the hospital stay. Hospital deaths refer to deaths during the hospital stay after discharge from the ICU admission where withholding or withdrawal orders were made.

# Figure S2

**Figure S2.** Flow diagram of patients for DNR order

*DNR* do-not-resuscitate, *ICU* intensive care unit

Given that we are measuring the secondary outcome of hospital mortality with patients who had DNR orders, numbers refer to patients rather than ICU admissions and readmissions. Patients are categorised as receiving DNR orders (a form of withholding) as long as the orders were made in at least one ICU admission during the hospital stay. ICU deaths refer to the ICU admission where DNR orders were made for patients with multiple ICU admissions within the hospital stay. Hospital deaths refer to deaths during the hospital stay after discharge from the ICU admission where DNR orders were made.

# Figure S3

**Figure S3.** Flow diagram of patients for invasive mechanical ventilation

*ICU* intensive care unit

Given the aim of comparing the secondary outcome of hospital mortality with the primary outcomes of withholding and withdrawal of life-sustaining treatments, numbers refer to patients rather than ICU admissions and readmissions. Patients are categorised as receiving withholding or withdrawal orders as long as the orders were made in at least one ICU admission during the hospital stay. ICU deaths refer to the ICU admission where withholding or withdrawal orders were made for patients with multiple ICU admissions within the hospital stay. Hospital deaths refer to deaths during the hospital stay after discharge from the ICU admission where withholding or withdrawal orders were made.

# Figure S4

**Figure S4**. Flow diagram of patients for vasopressors/inotropes

*ICU* intensive care unit

Given the aim of comparing the secondary outcome of hospital mortality with the primary outcomes of withholding and withdrawal of life-sustaining treatments, numbers refer to patients rather than ICU admissions and readmissions. Patients are categorised as receiving withholding or withdrawal orders as long as the orders were made in at least one ICU admission during the hospital stay. ICU deaths refer to the ICU admission where withholding or withdrawal orders were made for patients with multiple ICU admissions within the hospital stay. Hospital deaths refer to deaths during the hospital stay after discharge from the ICU admission where withholding or withdrawal orders were made.
